# New records of Ichneumonidae (Hymenoptera) for the Italian fauna

**DOI:** 10.3897/BDJ.3.e5057

**Published:** 2015-06-19

**Authors:** Filippo Di Giovanni, Alexey Reshchikov, Matthias Riedel, Erich Diller, Martin Schwarz

**Affiliations:** ‡ University of Rome "Sapienza", Rome, Italy; § Swedish Museum of Natural History, Stockholm, Sweden; | Amselweg 9 A, Bad Fallingbostel, Germany; ¶ Zoologische Staatssammlung München, Munich, Germany; # Biologiezentrum, Linz, Austria

**Keywords:** Ichneumonidae, Italy, checklist, Italian mainland, Sicily, Sardinia, new records

## Abstract

New distributional records on 55 ichneumonids (Hymenoptera, Ichneumonidae) from Italy are provided. Of these, 47 species are new for Italy, including representatives of the subfamily Diacritinae and of the tribes Zimmeriini (Ichneumoninae) and Pseudorhyssini (Poemeniinae); six species are new for Sardinia, one for Sicily and one for the Italian mainland. The hitherto unknown female of *Baranisobas hibericus* Heinrich, 1972 (Ichneumoninae) is described.

## Introduction

With more than 24,000 species described (Yu et al. 2012), Ichneumonidae is one of the largest families of Hymenoptera and one of the largest within insects. More than 6,500 species have been recorded for Europe so far (Achterberg and Zwakhals 2004). Despite their richness and the role they play as pest control agents, the faunistic knowledge of this group in many European countries and worldwide remains scarce.

It is difficult to give a precise estimate of the number of species of Ichneumonidae occurring in Italy. About 1,850 species are recorded for the country in the checklist of the Italian fauna (Scaramozzino 1995). Most of the data refer to northern and central regions of Italy, while records on the ichneumonid fauna of the south, Sicily and Sardinia are relatively few (Scaramozzino 1995, Zwakhals and Turrisi 2014). More recently, the Fauna Europaea project increased this number to about 2,000 species (Achterberg and Zwakhals 2004). In both cases, however, it is not possible to link records in the checklist to voucher specimens in collections or to data in the literature, which makes it difficult to verify the records, keep track of nomenclatorial and systematic changes, and update the checklist accordingly. Based exclusively on records from the literature, the Taxapad database (Yu et al. 2012) reports about 1,700 species for Italy, but the real number of species in the country is likely to be much higher. By crossing the references in the Italian checklist (Scaramozzino 1995) with those in Fauna Europaea and Taxapad (Achterberg and Zwakhals 2004, Yu et al. 2012), the number of ichneumonid species recorded for Italy reaches about 2,200 species. This result cannot be considered reliable, as only a direct verification of the literature and of the specimens in private and public collections can attest the real number and identity of ichneumonid species found in Italy. References to the literature and/or to voucher specimens in collections would be essential to make a scientific and reliable checklist, and would allow to track taxonomic changes through time and update the checklist properly.

The present study reports 47 new species for the Italian fauna, six new species for Sardinia, one for Sicily and one new record for the Italian mainland. Thanks to some intensive samplings conducted in Italy in the last few years, our paper provides a remarkable - though probably still incomplete - contribution to the knowledge of this group in the Italian territory.

## Materials and methods

**Figure 1.**
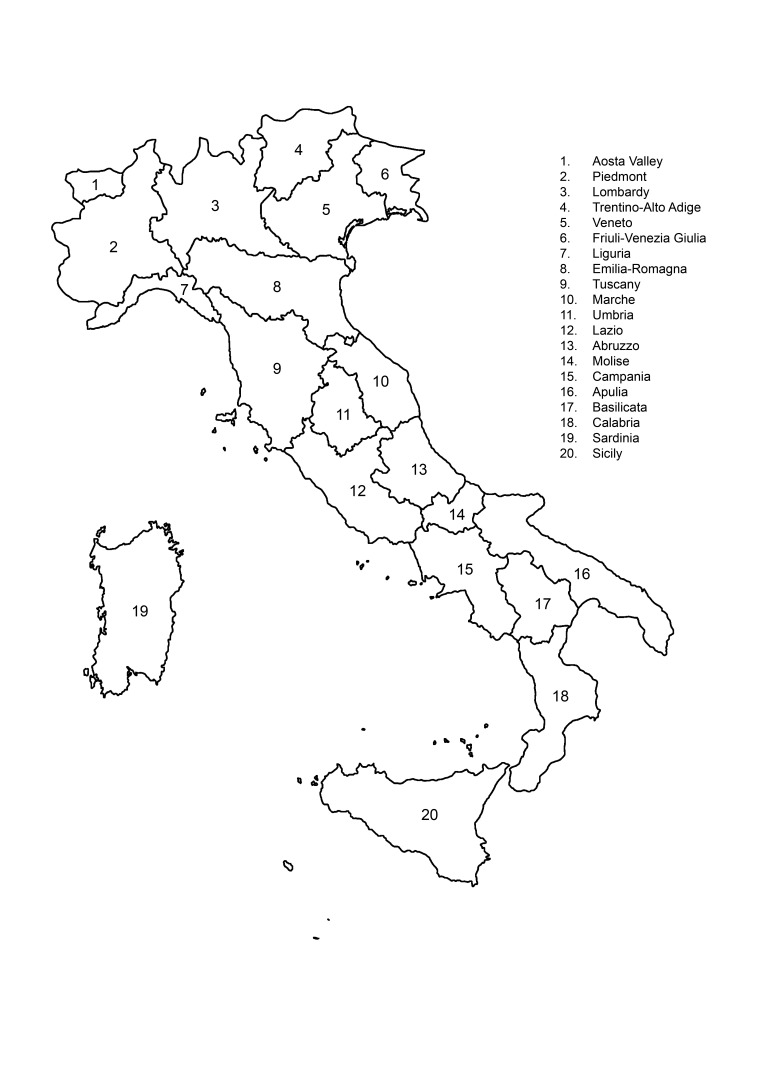
Italian administrative regions.

**Figure 2.**
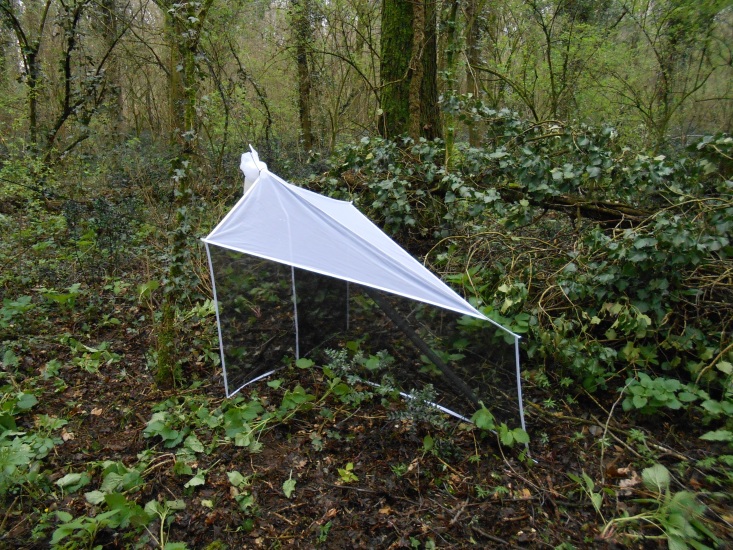
Malaise trap in an oak-hornbeam forest in north-eastern Italy.

**Figure 3.**
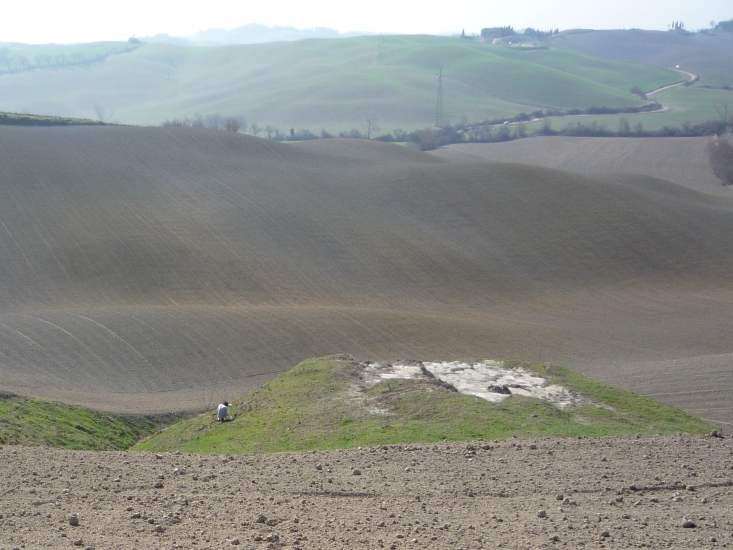
Field sampling with yellow pan traps in Tuscany claystones (courtesy of D. Inclán).

Most of the records in the present paper originate from sampling efforts in central and north-eastern Italy (Fig. 1) aimed at investigating parasitoid community composition in fragmented landscapes (Di Giovanni et al. 2014, Inclán et al. 2014). The field sampling in north-eastern Italy was carried out in 2013 using Malaise traps (Omnes Artes s. a. s.) in the understory of semi-natural and artificial oak-hornbeam forests of the eastern Po Plain (Fig. 2). Some further records stem from canopy sampling with Malaise traps, carried out in 2008 in the natural reserve of Bosco della Fontana (Lombardy; see Di Giovanni et al. 2014). The field sampling in central Italy (Tuscany) was conducted in 2012 using yellow pan traps in fragments of semi-natural grasslands derived from eroded claystones (Fig. 3; see Inclán et al. 2014). Additional records come from the private collection of the first author (with the exception of one specimen from DAFNAE and one from ZSM) and from the Sardinian CONECOFOR (Forest Ecosystem Control) sampling project, carried out by Italian State Forestry Service between 2003 and 2006 (see Nardi et al. 2011).

All taxa are listed in alphabetical order. The assignment of species to higher taxa follows the Catalogue of World Ichneumonidae (Yu et al. 2012). Distributional notes, with minor changes, follow the Catalogue of World Ichneumonidae (Yu et al. 2012) and the Fauna Europaea website (Achterberg and Zwakhals 2004).

The following references were used to the identification of most of the species listed in the present paper. It is not an exhaustive list, as many genera still need a revision and the identification to species level often requires the comparison with type material in collections: Varga (2013) (Acaenitinae); Kasparyan (1990) (Adelognathinae); Kuslitzky (1973), Aubert (1978) (Banchinae); Horstmann (2009) (Campopleginae); Gürbüz and Kolarov (2006) (Collyriinae); Donovan (1810), Förster (1871), Habermehl (1935), Horstmann (1983), Townes (1983), Horstmann (1990) (Cryptinae); Gravenhorst (1829), Bauer (1961), Kasparyan (1981), Hinz (1986), Hinz (1991), Hinz and Horstmann (1998), Kasparyan (2000), Kasparyan (2001), Kasparyan (2004), Kasparyan (2011), Reshchikov (2013), Kasparyan (2014) (Ctenopelmatinae); Kolarov (1997) (Diacritinae); Klopfstein (2014) (Diplazontinae); Aubert (1958), Aubert (1969), Kasparyan (1981), Selfa and Diller (1995), Selfa and Anento (1996), Tereshkin (2009), Diller and Shaw (2014) (Ichneumoninae); Kasparyan (1981) (Lycorininae); Schwenke (1999) (Mesochorinae); Aubert (1965) (Metopiinae); Broad (2010) (Orthocentrinae); Kolarov (1997), Zwakhals (2006), Fritzén and Shaw (2014) (Pimplinae); Oehlke (1966), Kolarov (1997) (Poemeniinae); Kasparyan (1973) (Tryphoninae).

The material is deposited in the following collections:

- DAFNAE: Dipartimento di Agronomia, Animali, Alimenti, Risorse Naturali e Ambiente in Padua, Italy
- MR: private collection of Matthias Riedel in Bad Fallingbostel, Germany
- MZUR: Museo di Zoologia, Università degli Studi di Roma “Sapienza” in Rome, Italy
- NRM: Naturhistoriska Riksmuseet in Stockholm, Sweden
- ZSM: Zoologische Staatssammlung in München, Germany

## Taxon treatments

### Baranisobas hibericus

Heinrich, 1972

#### Materials

- **Type status:** Other material. **Occurrence:** recordedBy: D. J. Inclán; individualCount: 1; sex: female; **Location:** country: Italy; stateProvince: Tuscany; verbatimLocality: Siena, Monteroni d'Arbia; verbatimElevation: 195 m; verbatimLatitude: 43°13'5.48"N; verbatimLongitude: 11°26'55.29"E; **Identification:** identifiedBy: M. Riedel; dateIdentified: 2014; **Event:** samplingProtocol: yellow pan trap; eventDate: 02-05.V.2012; **Record Level:** institutionCode: MR
- **Type status:** Other material. **Occurrence:** recordedBy: D. J. Inclán; individualCount: 1; sex: female; **Location:** country: Italy; stateProvince: Tuscany; verbatimLocality: Siena, Monteroni d'Arbia; verbatimElevation: 195 m; verbatimLatitude: 43°13'5.48"N; verbatimLongitude: 11°26'55.29"E; **Identification:** identifiedBy: M. Riedel; dateIdentified: 2014; **Event:** samplingProtocol: yellow pan trap; eventDate: 11-12.V.2012; **Record Level:** institutionCode: MR

#### Description

**Male** (from Heinrich 1972):

Mesoscutum with dense and rather coarse punctures, ground between punctures smooth and shiny. Margin of clypeus shiny. Malar space about ¾ the length of the base of mandible. Flagellum with short elliptical tyloids on flagellomere 7-16.

Colour: face and thorax mostly black; white are inner orbits, narrow stripe on outer orbits, central spot on face, scapus and flagellum ventrally, collar, upper hind margin of pronotum, subalar ridge, tegulae, scutellum and postscutellum. Metasoma black; postpetiolus, tergite 2 and 3, and basal part of tergite 4 red; tergite 6 with white spot, tergite 7 mostly white. Coxae, trochanters and legs black; coxae I and II with white spot, ventral side of tibiae I and II, femur I except the base and apex of femur II pale yellowish.

**Female:**

Body length 6-7 mm. Flagellum with 27-28 segments, slightly lanceolate. First flagellomere about 1.8 times longer than wide, widest flagellomeres about 1.2 times wider than long. Temples roundly narrowed behind eyes. Clypeus moderately convex. Head with coarse dense puncture, clypeus with scattered puncture.

Pronotum with a strong longitudinal ridge medially. Mesoscutum with coarse puncture and fine granulation, strongly shining. Mesopleurum with coarse puncture (slightly rugose-punctate apically), intervals smooth and shining, metapleurum with coarse puncture, shining. Scutellum slightly wider than long, with lateral carinae in the basal 0.5. Propodeum completely carinate, area superomedia hexagonal, about as long as wide. Hind coxa punctate, without scopa. Hind femur stout, about 3.3 times as long as wide.

Postpetiolus strongly widened, not differentiate and without dorsal carina, almost smooth and with very scattered puncture medially. Second tergite with strong transverse thyridia, each thyridium about 1.6 times wider than the thyridial interval. Tergites 2-3 with coarse puncture, shining, tergite 4 superficially punctate, following tergites smoothened.

Colour: mostly black. Flagellum with ivory ring on flagellomeres 7-11. Ivory are narrow stripe on frontal orbit, apical half of scutellum, narrow apical band on tergite 6 (one specimen with completely black tergite 6) and wide apical band on tergite 7. Tergites 1-3 red. Following tergites black, the fourth one more or less reddish laterally. Coxae and trochanteres black. Femora I-II reddish, more or less infuscate at bases. Tibiae and tarsi I-II reddish, yellowish-red on frontal side. Hind leg black, tibia III with red subbasal ring. Pterostigma black.

#### Diagnosis

**Figure 4.**
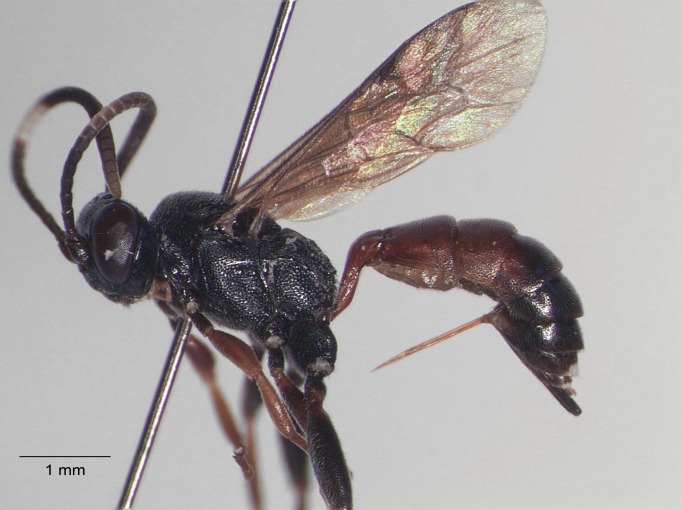
*Baranisobas hibericus* Heinrich, 1972, female habitus.

**Figure 5.**
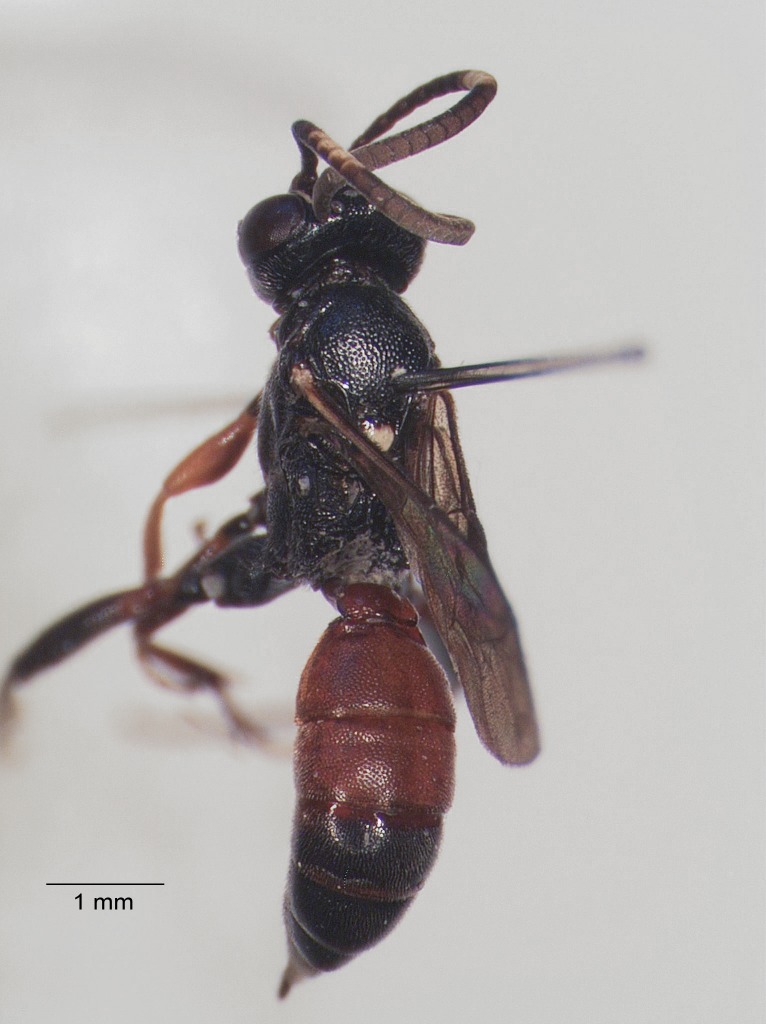
*Baranisobas hibericus* Heinrich, 1972, female, dorsal view.

The female of *B. hibericus* Heinrich is similar to *B. ridibundus* (Gravenhorst, 1829) but it differs in having a stouter first flagellomere, strongly shining mesoscutum with coarse punctation, and a stouter hind femur (Figs 4, 5​).

#### Distribution

Previously known only from Portugal (Heinrich 1972​).

#### Notes

New for Italy.

## Checklists

### Species new for Italy

#### Acaenitinae

Förster, 1869

#### Coleocentrini

Clément, 1938

#### Coleocentrus heteropus

Thomson, 1894

##### Materials

- **Type status:** Other material. **Occurrence:** recordedBy: D. Sechi; individualCount: 1; sex: female; **Location:** country: Italy; stateProvince: Trentino-Alto Adige; verbatimLocality: Bolzano, Castelrotto, Alpe di Marinzen; **Identification:** identifiedBy: F. Di Giovanni; dateIdentified: 2014; **Event:** eventDate: 13.VII.2013; **Record Level:** institutionCode: MZUR

##### Distribution

Finland, Sweden, Hungary, Romania and Ukraine (Varga 2013​).

##### Notes

New for Italy.

#### Adelognathinae

Thomson, 1888

#### Adelognathus maculosus

Kasparyan, 1990

##### Materials

- **Type status:** Other material. **Occurrence:** recordedBy: G. Chessa; individualCount: 1; sex: female; **Location:** country: Italy; stateProvince: Sardinia; verbatimLocality: Iglesias, Marganai; verbatimElevation: 700 m; verbatimLatitude: 39°20'59.74"N; verbatimLongitude: 8°34'18.70"E; **Identification:** identifiedBy: A. Reshchikov; dateIdentified: 2015; **Event:** samplingProtocol: Malaise trap; eventDate: 20.V-16.VI.2005; **Record Level:** institutionCode: NRM

##### Distribution

South Europe and Middle East.

##### Notes

Already recorded for Italy. It is new for Sardinia.

#### Banchinae

Wesmael, 1845

#### Glyptini

Cushman & Rohwer, 1920

#### Glypta cylindrator

(Fabricius, 1787)

##### Materials

- **Type status:** Other material. **Occurrence:** recordedBy: M. Romano; individualCount: 1; sex: male; **Location:** country: Italy; stateProvince: Sicily; verbatimLocality: Palermo, Madonie, Vallone di Zottafonda; verbatimElevation: 1600 m; **Identification:** identifiedBy: F. Di Giovanni; dateIdentified: 2013; **Event:** eventDate: 20.VI.1998; **Record Level:** institutionCode: MZUR

##### Distribution

Palaearctic.

##### Notes

Already recorded for Italy. It is new for Sicily.

#### Teleutaea striata

(Gravenhorst, 1829)

##### Materials

- **Type status:** Other material. **Occurrence:** recordedBy: F. Di Giovanni; individualCount: 1; sex: male; **Location:** country: Italy; stateProvince: Veneto; verbatimLocality: Venezia, Mestre, bosco di Zaher; verbatimElevation: 0 m; verbatimLatitude: 45°31'13.70"N; verbatimLongitude: 12°17'20.71"E; **Identification:** identifiedBy: F. Di Giovanni; dateIdentified: 2014; **Event:** samplingProtocol: Malaise trap; eventDate: 22.VII-04.VIII.2013; **Record Level:** institutionCode: MZUR

##### Distribution

Palaearctic.

##### Notes

**Figure 6.**
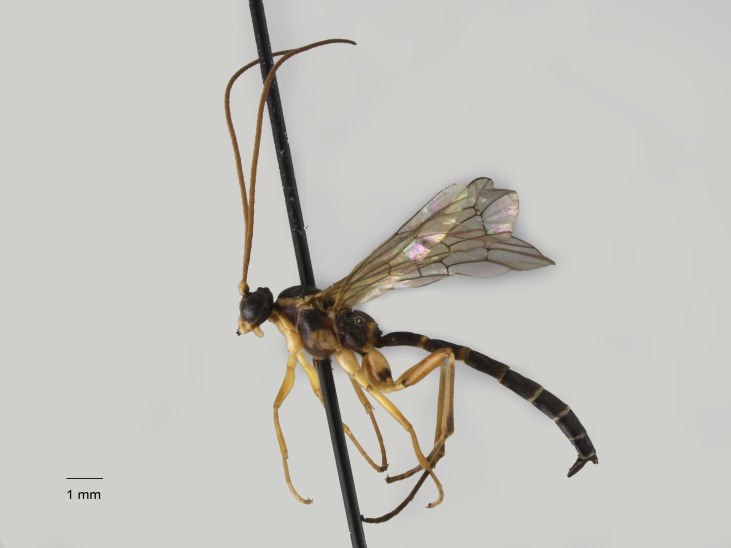
*Teleutaea striata* (Gravenhorst, 1829), male.

New for Italy (​Fig. 6​).

#### Campopleginae

Förster, 1869

#### Dusona aurita

(Kriechbaumer, 1883)

##### Materials

- **Type status:** Other material. **Occurrence:** recordedBy: F. Di Giovanni; individualCount: 1; sex: female; **Location:** country: Italy; stateProvince: Friuli-Venezia Giulia; verbatimLocality: Udine, Palazzolo dello Stella, Nogali Braide, bosco Brussa; verbatimElevation: 0 m; verbatimLatitude: 45°45'54.05"N; verbatimLongitude: 13°04'52.15"E; **Identification:** identifiedBy: F. Di Giovanni; dateIdentified: 2014; **Event:** samplingProtocol: Malaise trap; eventDate: 09-21.VI.2013; **Record Level:** institutionCode: MZUR
- **Type status:** Other material. **Occurrence:** recordedBy: F. Di Giovanni; individualCount: 1; sex: female; **Location:** country: Italy; stateProvince: Veneto; verbatimLocality: Treviso, Gaiarine, Francenigo, bosco Otello; verbatimElevation: 20 m; verbatimLatitude: 45°51'38.06"N; verbatimLongitude: 12°29'31.71"E; **Identification:** identifiedBy: F. Di Giovanni; dateIdentified: 2014; **Event:** samplingProtocol: Malaise trap; eventDate: 23.VII-05.VIII.2013; **Record Level:** institutionCode: MZUR

##### Distribution

Palaearctic.

##### Notes

New for Italy.

#### Collyriinae

Cushman, 1924

#### Collyria trichophthalma

(Thomson, 1877)

##### Materials

- **Type status:** Other material. **Occurrence:** recordedBy: F. Di Giovanni; individualCount: 6; sex: 2 males, 4 females; **Location:** country: Italy; stateProvince: Friuli-Venezia Giulia; verbatimLocality: Udine, Marano Lagunare, proprietà Villabruna; verbatimElevation: 0 m; verbatimLatitude: 45°46'36.36"N; verbatimLongitude: 13°09'32.06"E; **Identification:** identifiedBy: F. Di Giovanni; dateIdentified: 2013; **Event:** samplingProtocol: Malaise trap; eventDate: 08-18.V.2013; **Record Level:** institutionCode: MZUR

##### Distribution

Europe.

##### Notes

New for Italy.

#### Cryptinae

Kirby, 1837

#### Cryptini

Kirby, 1837

#### Agrothereutes leucorhaeus

(Donovan, 1810)

##### Materials

- **Type status:** Other material. **Occurrence:** recordedBy: D. J. Inclán; individualCount: 1; sex: female; **Location:** country: Italy; stateProvince: Tuscany; verbatimLocality: Siena, Chiusure; verbatimElevation: 340 m; verbatimLatitude: 43°10'53.96"N; verbatimLongitude: 11°34'48.71"E; **Identification:** identifiedBy: M. Schwarz; dateIdentified: 2013; **Event:** samplingProtocol: yellow pan trap; eventDate: 26-27.V.2012; **Record Level:** institutionCode: MZUR

##### Distribution

Europe.

##### Notes

New for Italy.

#### Agrothereutes monticola

(Habermehl, 1935)

##### Materials

- **Type status:** Other material. **Occurrence:** recordedBy: D. J. Inclán; individualCount: 1; sex: female; **Location:** country: Italy; stateProvince: Tuscany; verbatimLocality: Siena, Torrenieri; verbatimElevation: 220 m; verbatimLatitude: 43°07'19.96"N; verbatimLongitude: 11°33'26.49"E; **Identification:** identifiedBy: M. Schwarz; dateIdentified: 2013; **Event:** samplingProtocol: yellow pan trap; eventDate: 22-23.VI.2012; **Record Level:** institutionCode: MZUR

##### Distribution

Previously known only from Spain and France.

##### Notes

**Figure 7.**
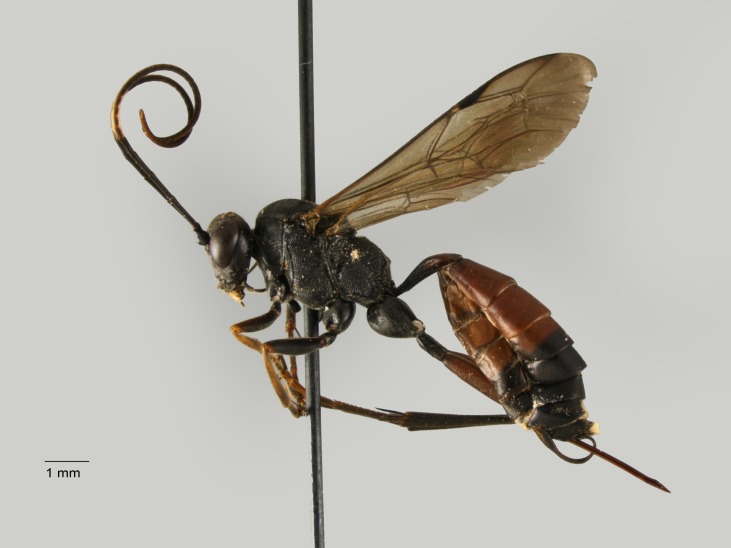
*Agrothereutes monticola* (Habermehl, 1935), female.

New for Italy (Fig. 7​)

#### Ateleute linearis

Förster, 1871

##### Materials

- **Type status:** Other material. **Occurrence:** recordedBy: F. Di Giovanni; individualCount: 1; sex: female; **Location:** country: Italy; stateProvince: Veneto; verbatimLocality: Treviso, Cessalto, Santa Maria di Campagna, bosco San Marco; verbatimElevation: 0 m; verbatimLatitude: 45°42'21.22"N; verbatimLongitude: 12°34'42.45"E; **Identification:** identifiedBy: F. Di Giovanni; dateIdentified: 2013; **Event:** samplingProtocol: Malaise trap; eventDate: 23.VII-05.VIII.2013; **Record Level:** institutionCode: MZUR
- **Type status:** Other material. **Occurrence:** recordedBy: F. Di Giovanni; individualCount: 1; sex: female; **Location:** country: Italy; stateProvince: Veneto; verbatimLocality: Treviso, Meolo; verbatimElevation: 0 m; verbatimLatitude: 45°36'24.76"N; verbatimLongitude: 12°27'25.19"E; **Identification:** identifiedBy: F. Di Giovanni; dateIdentified: 2013; **Event:** samplingProtocol: Malaise trap; eventDate: 23.VII-05.VIII.2013; **Record Level:** institutionCode: MZUR

##### Distribution

Europe.

##### Notes

New for Italy.

#### Phygadeuontini

Förster, 1869

#### Chirotica rubrotincta

(Thomson, 1885)

##### Materials

- **Type status:** Other material. **Occurrence:** recordedBy: G. Lo Giudice, M. Mei, D. Petriccione; individualCount: 1; sex: female; **Location:** country: Italy; stateProvince: Latium; verbatimLocality: Rieti, Orvinio, Vallebuona; verbatimElevation: 780 m; **Identification:** identifiedBy: F. Di Giovanni; dateIdentified: 2012; **Event:** eventDate: 15.VI.2008; **Record Level:** institutionCode: MZUR

##### Distribution

South Europe and North Africa.

##### Notes

New for Italy.

#### Mastrus rufobasalis

(Habermehl, 1920)

##### Materials

- **Type status:** Other material. **Occurrence:** recordedBy: D. J. Inclán; individualCount: 1; sex: female; **Location:** country: Italy; stateProvince: Tuscany; verbatimLocality: Siena, Pienza; verbatimElevation: 335 m; verbatimLatitude: 43°03'51.89"N; verbatimLongitude: 11°39'56.44"E; **Identification:** identifiedBy: M. Schwarz; dateIdentified: 2013; **Event:** samplingProtocol: yellow pan trap; eventDate: 02-05.X.2012; **Record Level:** institutionCode: MZUR

##### Distribution

Europe.

##### Notes

New for Italy.

#### Xenolytus substriatus

Townes, 1983

##### Materials

- **Type status:** Other material. **Occurrence:** recordedBy: D. J. Inclán; individualCount: 1; sex: female; **Location:** country: Italy; stateProvince: Tuscany; verbatimLocality: Siena, Arbia; verbatimElevation: 220 m; verbatimLatitude: 43°16'53.18"N; verbatimLongitude: 11°25'35.12"E; **Identification:** identifiedBy: M. Schwarz; dateIdentified: 2013; **Event:** samplingProtocol: yellow pan trap; eventDate: 02-05.X.2012; **Record Level:** institutionCode: MZUR
- **Type status:** Other material. **Occurrence:** recordedBy: D. J. Inclán; individualCount: 1; sex: female; **Location:** country: Italy; stateProvince: Tuscany; verbatimLocality: Siena, Bollano; verbatimElevation: 230 m; verbatimLatitude: 43°10'32.29"N; verbatimLongitude: 11°31'44.98"E; **Identification:** identifiedBy: M. Schwarz; dateIdentified: 2013; **Event:** samplingProtocol: yellow pan trap; eventDate: 02-05.X.2012; **Record Level:** institutionCode: MZUR
- **Type status:** Other material. **Occurrence:** recordedBy: D. J. Inclán; individualCount: 1; sex: female; **Location:** country: Italy; stateProvince: Tuscany; verbatimLocality: Siena, Arbia; verbatimElevation: 220 m; verbatimLatitude: 43°16'53.18"N; verbatimLongitude: 11°25'35.12"E; **Identification:** identifiedBy: M. Schwarz; dateIdentified: 2013; **Event:** samplingProtocol: yellow pan trap; eventDate: 23-26.X.2012; **Record Level:** institutionCode: MZUR

##### Distribution

Europe.

##### Notes

New for Italy.

#### Ctenopelmatinae

Förster, 1869

#### Euryproctini

Thomson, 1883

#### Hadrodactylus nigrifemur

Thomson, 1883

##### Materials

- **Type status:** Other material. **Occurrence:** recordedBy: F. Di Giovanni; individualCount: 1; sex: female; **Location:** country: Italy; stateProvince: Veneto; verbatimLocality: Venezia, Mestre, bosco di Zaher; verbatimElevation: 0 m; verbatimLatitude: 45°31'13.70"N; verbatimLongitude: 12°17'20.71"E; **Identification:** identifiedBy: A. Reshchikov; dateIdentified: 2014; **Event:** samplingProtocol: Malaise trap; eventDate: 09-19.V.2013; **Record Level:** institutionCode: MZUR
- **Type status:** Other material. **Occurrence:** recordedBy: F. Di Giovanni; individualCount: 1; sex: female; **Location:** country: Italy; stateProvince: Veneto; verbatimLocality: Venezia, Mestre, bosco di Zaher; verbatimElevation: 0 m; verbatimLatitude: 45°31'13.70"N; verbatimLongitude: 12°17'20.71"E; **Identification:** identifiedBy: A. Reshchikov; dateIdentified: 2014; **Event:** samplingProtocol: Malaise trap; eventDate: 09-19.V.2013; **Record Level:** institutionCode: NRM

##### Distribution

Palaearctic.

##### Notes

New for Italy.

#### Phobetes nigriventris

(Teunissen, 1953)

##### Materials

- **Type status:** Other material. **Occurrence:** recordedBy: F. Di Giovanni; individualCount: 2; sex: males; **Location:** country: Italy; stateProvince: Friuli-Venezia Giulia; verbatimLocality: Udine, San Giorgio di Nogaro, bosco Ronchi di Sass; verbatimElevation: 5 m; verbatimLatitude: 45°48'21.10"N; verbatimLongitude: 13°14'27.08"E; **Identification:** identifiedBy: A. Reshchikov; dateIdentified: 2014; **Event:** samplingProtocol: Malaise trap; eventDate: 08-18.V.2013; **Record Level:** institutionCode: MZUR
- **Type status:** Other material. **Occurrence:** recordedBy: F. Di Giovanni; individualCount: 2; sex: 1 male, 1 female; **Location:** country: Italy; stateProvince: Veneto; verbatimLocality: Treviso, Mansuè, bosco di Basalghelle; verbatimElevation: 25 m; verbatimLatitude: 45°49'44.28"N; verbatimLongitude: 12°31'12.07"E; **Identification:** identifiedBy: A. Reshchikov; dateIdentified: 2014; **Event:** samplingProtocol: Malaise trap; eventDate: 09-19.V.2013; **Record Level:** institutionCode: NRM
- **Type status:** Other material. **Occurrence:** recordedBy: F. Di Giovanni; individualCount: 1; sex: male; **Location:** country: Italy; stateProvince: Veneto; verbatimLocality: Treviso, Gaiarine, Francenigo, bosco Otello; verbatimElevation: 20 m; verbatimLatitude: 45°51'38.06"N; verbatimLongitude: 12°29'31.71"E; **Identification:** identifiedBy: A. Reshchikov; dateIdentified: 2014; **Event:** samplingProtocol: Malaise trap; eventDate: 09-19.V.2013; **Record Level:** institutionCode: MZUR
- **Type status:** Other material. **Occurrence:** recordedBy: F. Di Giovanni; individualCount: 3; sex: males; **Location:** country: Italy; stateProvince: Lombardy; verbatimLocality: Mantova, Marmirolo, Bosco della Fontana; verbatimElevation: 35 m; verbatimLatitude: 45°12'00.16"N; verbatimLongitude: 10°44'38.78"E; **Identification:** identifiedBy: A. Reshchikov; dateIdentified: 2014; **Event:** samplingProtocol: Malaise trap; eventDate: 11-21.V.2013; **Record Level:** institutionCode: MZUR
- **Type status:** Other material. **Occurrence:** recordedBy: F. Di Giovanni; individualCount: 1; sex: male; **Location:** country: Italy; stateProvince: Friuli-Venezia Giulia; verbatimLocality: Udine, San Giorgio di Nogaro, bosco Ronchi di Sass; verbatimElevation: 5 m; verbatimLatitude: 45°48'21.10"N; verbatimLongitude: 13°14'27.08"E; **Identification:** identifiedBy: A. Reshchikov; dateIdentified: 2014; **Event:** samplingProtocol: Malaise trap; eventDate: 09-21.VI.2013; **Record Level:** institutionCode: MZUR
- **Type status:** Other material. **Occurrence:** recordedBy: F. Di Giovanni; individualCount: 2; sex: males; **Location:** country: Italy; stateProvince: Friuli-Venezia Giulia; verbatimLocality: Udine, Carlino, bosco Pro Quain e Venchiarate; verbatimElevation: 5 m; verbatimLatitude: 45°47'06.26"N; verbatimLongitude: 13°12'54.68"E; **Identification:** identifiedBy: A. Reshchikov; dateIdentified: 2014; **Event:** samplingProtocol: Malaise trap; eventDate: 09-21.VI.2013; **Record Level:** institutionCode: MZUR

##### Distribution

 Previously recorded only for Netherlands and Russia.

##### Notes

**Figure 8.**
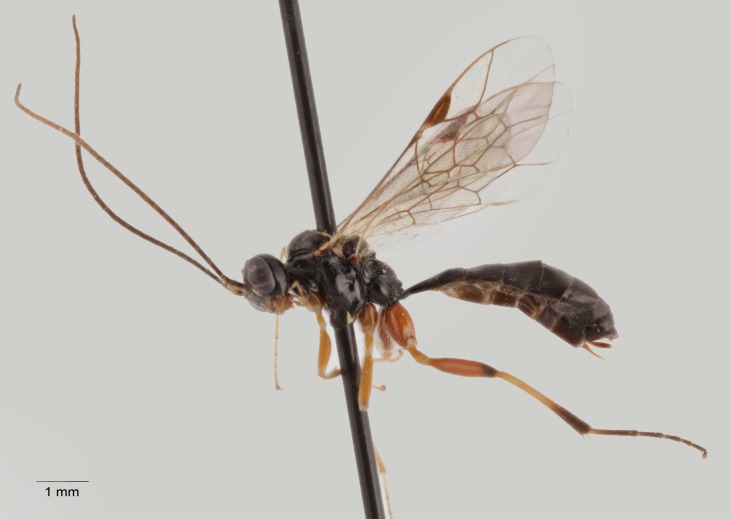
*Phobetes nigriventris* (Teunissen, 1953), female.

New for Italy (Fig. 8​​​).

#### Mesoleiini

Thomson, 1883

#### Mesoleius aulicus

(Gravenhorst, 1829)

##### Materials

- **Type status:** Other material. **Occurrence:** recordedBy: G. Chessa; individualCount: 1; sex: female; **Location:** country: Italy; stateProvince: Sardinia; verbatimLocality: Iglesias, dintorni colonia Beneck; verbatimElevation: 636 m; verbatimLatitude: 39°20'51.45"N; verbatimLongitude: 8°33'55.40"E; **Identification:** identifiedBy: A. Reshchikov; dateIdentified: 2015; **Event:** samplingProtocol: Malaise trap; eventDate: 02-16.V.2006; **Record Level:** institutionCode: NRM

##### Distribution

Holarctic.

##### Notes

Already recorded for Italy. It is new for Sardinia.

#### Mesoleius tibialis

Holmgren, 1857

##### Materials

- **Type status:** Other material. **Occurrence:** recordedBy: G. Chessa; individualCount: 2; sex: females; **Location:** country: Italy; stateProvince: Sardinia; verbatimLocality: Iglesias, dintorni colonia Beneck; verbatimElevation: 636 m; verbatimLatitude: 39°20'51.45"N; verbatimLongitude: 8°33'55.40"E; **Identification:** identifiedBy: A. Reshchikov; dateIdentified: 2015; **Event:** samplingProtocol: Malaise trap; eventDate: 02-16.V.2006; **Record Level:** institutionCode: NRM

##### Distribution

Europe.

##### Notes

Already recorded for Italy. It is new for Sardinia.

#### Mesoleius tibiator

Kasparyan, 2000

##### Materials

- **Type status:** Other material. **Occurrence:** recordedBy: F. Di Giovanni; individualCount: 1; sex: female; **Location:** country: Italy; stateProvince: Friuli-Venezia Giulia; verbatimLocality: Udine, Muzzana del Turgnano; verbatimElevation: 0 m; verbatimLatitude: 45°47'56.80"N; verbatimLongitude: 13°06'36.09"E; **Identification:** identifiedBy: A. Reshchikov; dateIdentified: 2014; **Event:** samplingProtocol: Malaise trap; eventDate: 21.VII-03.VIII.2013; **Record Level:** institutionCode: NRM

##### Distribution

Palaearctic.

##### Notes

**Figure 9.**
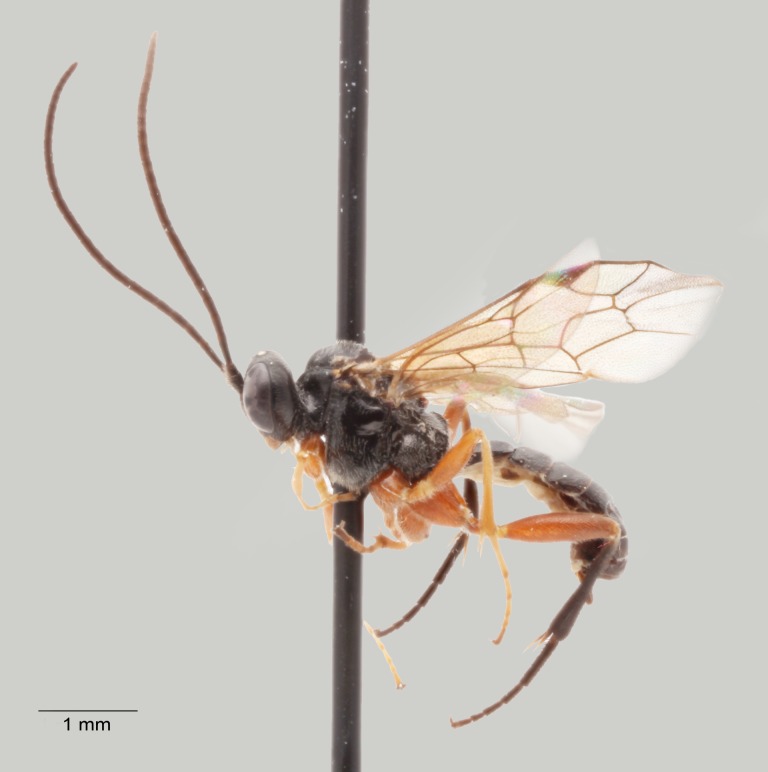
*Mesoleius tibiator* Kasparyan, 2000, female.

New for Italy (Fig. 9​​​). In Europe, it is known from Scandinavia (Kasparyan 2000​​). This is the most southerly record for this species.

#### Rhinotorus leucostomus

(Gravenhorst, 1829)

##### Materials

- **Type status:** Other material. **Occurrence:** recordedBy: F. Di Giovanni; individualCount: 1; sex: female; **Location:** country: Italy; stateProvince: Veneto; verbatimLocality: Treviso, Meolo; verbatimElevation: 0 m; verbatimLatitude: 45°36'24.76"N; verbatimLongitude: 12°27'25.19"E; **Identification:** identifiedBy: A. Reshchikov; dateIdentified: 2014; **Event:** samplingProtocol: Malaise trap; eventDate: 10-22.VI.2013; **Record Level:** institutionCode: NRM
- **Type status:** Other material. **Occurrence:** recordedBy: F. Di Giovanni; individualCount: 1; sex: female; **Location:** country: Italy; stateProvince: Friuli-Venezia Giulia; verbatimLocality: Udine, Marano Lagunare; verbatimElevation: 0 m; verbatimLatitude: 45°46'36.36"N; verbatimLongitude: 13°09'32.06"E; **Identification:** identifiedBy: A. Reshchikov; dateIdentified: 2014; **Event:** samplingProtocol: Malaise trap; eventDate: 21.VII-03.VIII.2013; **Record Level:** institutionCode: NRM
- **Type status:** Other material. **Occurrence:** recordedBy: F. Di Giovanni; individualCount: 1; sex: female; **Location:** country: Italy; stateProvince: Veneto; verbatimLocality: Treviso, Meolo; verbatimElevation: 0 m; verbatimLatitude: 45°36'24.76"N; verbatimLongitude: 12°27'25.19"E; **Identification:** identifiedBy: A. Reshchikov; dateIdentified: 2014; **Event:** samplingProtocol: Malaise trap; eventDate: 23.VII-05.VIII.2013; **Record Level:** institutionCode: NRM

##### Distribution

Europe.

##### Notes

New for Italy.

#### Perilissini

Thomson, 1883

#### Absyrtus vernalis

Bauer, 1961

##### Materials

- **Type status:** Other material. **Occurrence:** recordedBy: F. Di Giovanni; individualCount: 1; sex: male; **Location:** country: Italy; stateProvince: Veneto; verbatimLocality: Treviso, Gaiarine, Francenigo, bosco Otello; verbatimElevation: 20 m; verbatimLatitude: 45°51'38.06"N; verbatimLongitude: 12°29'31.71"E; **Identification:** identifiedBy: A. Reshchikov; dateIdentified: 2014; **Event:** samplingProtocol: Malaise trap; eventDate: 09-19.V.2013; **Record Level:** institutionCode: NRM

##### Distribution

Europe.

##### Notes

New for Italy.

#### Lathrolestes (Lathrolestes) luteolator

(Gravenhorst, 1829)

##### Materials

- **Type status:** Other material. **Occurrence:** individualCount: 1; sex: male; **Location:** country: Italy; stateProvince: Veneto; verbatimLocality: Padova; **Identification:** identifiedBy: J. F. Perkins; dateIdentified: 1959; **Event:** eventDate: 17.V.1958; **Record Level:** institutionCode: DAFNAE
- **Type status:** Other material. **Occurrence:** occurrenceRemarks: collected in the canopy (15-20 m); individualCount: 1; sex: male; **Location:** country: Italy; stateProvince: Lombardy; verbatimLocality: Mantova, Marmirolo, Bosco della Fontana; verbatimElevation: 35 m; verbatimLatitude: 45°12'00.16"N; verbatimLongitude: 10°44'38.78"E; **Identification:** identifiedBy: A. Reshchikov; dateIdentified: 2014; **Event:** samplingProtocol: Malaise trap; eventDate: 13-27.V.2008; **Record Level:** institutionCode: NRM
- **Type status:** Other material. **Occurrence:** occurrenceRemarks: collected in the canopy (15-20 m); individualCount: 1; sex: female; **Location:** country: Italy; stateProvince: Lombardy; verbatimLocality: Mantova, Marmirolo, Bosco della Fontana; verbatimElevation: 35 m; verbatimLatitude: 45°12'00.16"N; verbatimLongitude: 10°44'38.78"E; **Identification:** identifiedBy: A. Reshchikov; dateIdentified: 2014; **Event:** samplingProtocol: Malaise trap; eventDate: 05-19.VIII.2008; **Record Level:** institutionCode: NRM

##### Distribution

Holarctic. Introduced into New Zealand.

##### Notes

The species was not recorded in the checklist of the Italian fauna (Scaramozzino 1995), even though a specimen of this species was present in the DAFNAE collection (Padua) and identified by Dr. J. F. Perkins in 1958. It is new for Italy.

#### Lathrolestes (Lathrolestes) tripunctor

(Thunberg, 1822)

##### Materials

- **Type status:** Other material. **Occurrence:** recordedBy: F. Di Giovanni; individualCount: 1; sex: female; **Location:** country: Italy; stateProvince: Friuli-Venezia Giulia; verbatimLocality: Udine, Palazzolo dello Stella, Nogali Braide, bosco Brussa; verbatimElevation: 0 m; verbatimLatitude: 45°45'54.05"N; verbatimLongitude: 13°04'52.15"E; **Identification:** identifiedBy: A. Reshchikov; dateIdentified: 2014; **Event:** samplingProtocol: Malaise trap; eventDate: 08-18.V.2013; **Record Level:** institutionCode: NRM

##### Distribution

Europe.

##### Notes

New for Italy.

#### Perilissus pallidus

(Gravenhorst, 1829)

##### Materials

- **Type status:** Other material. **Occurrence:** recordedBy: G. Chessa; individualCount: 1; sex: male; **Location:** country: Italy; stateProvince: Sardinia; verbatimLocality: Iglesias, dintorni colonia Beneck; verbatimElevation: 636 m; verbatimLatitude: 39°20'51.45"N; verbatimLongitude: 8°33'55.40"E; **Identification:** identifiedBy: A. Reshchikov; dateIdentified: 2015; **Event:** samplingProtocol: Malaise trap; eventDate: 02-16.V.2006; **Record Level:** institutionCode: NRM

##### Distribution

Palaearctic.

##### Notes

Already recorded for Italy. It is new for Sardinia.

#### Trematopygodes aprilinus

(Giraud, 1872)

##### Materials

- **Type status:** Other material. **Occurrence:** recordedBy: P. Cerretti, F. Di Giovanni, G. Lo Giudice; individualCount: 1; sex: female; **Location:** country: Italy; stateProvince: Veneto; verbatimLocality: Verona, dintorni di Monte, Stramonte; verbatimElevation: 500 m; **Identification:** identifiedBy: A. Reshchikov; dateIdentified: 2014; **Event:** eventDate: 05.V.2014; **Record Level:** institutionCode: NRM

##### Distribution

Europe.

##### Notes

New for Italy.

#### Pionini

Smith & Shenefelt, 1955

#### Rhorus nigrinus

(Habermehl, 1909)

##### Materials

- **Type status:** Other material. **Occurrence:** recordedBy: F. Di Giovanni; individualCount: 1; sex: male; **Location:** country: Italy; stateProvince: Veneto; verbatimLocality: Treviso, Cessalto, Santa Maria di Campagna, bosco San Marco; verbatimElevation: 0 m; verbatimLatitude: 45°42'21.22"N; verbatimLongitude: 12°34'42.45"E; **Identification:** identifiedBy: A. Reshchikov; dateIdentified: 2014; **Event:** samplingProtocol: Malaise trap; eventDate: 09-19.V.2013; **Record Level:** institutionCode: NRM

##### Distribution

Europe.

##### Notes

New for Italy.

#### Sympherta facialis

Hinz, 1991

##### Materials

- **Type status:** Other material. **Occurrence:** recordedBy: D. J. Inclán; individualCount: 1; sex: male; **Location:** country: Italy; stateProvince: Tuscany; verbatimLocality: Siena, Torrenieri; verbatimElevation: 220 m; verbatimLatitude: 43°07'19.96"N; verbatimLongitude: 11°33'26.49"E; **Identification:** identifiedBy: A. Reshchikov; dateIdentified: 2014; **Event:** samplingProtocol: yellow pan trap; eventDate: 27-28.IV.2012; **Record Level:** institutionCode: NRM

##### Distribution

Europe.

##### Notes

New for Italy.

#### Trematopygus lethierryi

Thomson, 1894

##### Materials

- **Type status:** Other material. **Occurrence:** recordedBy: L. Colacurcio; individualCount: 1; sex: male; **Location:** country: Italy; stateProvince: Emilia-Romagna; verbatimLocality: Bologna, Sasso Marconi, Palazzo Rossi; **Identification:** identifiedBy: A. Reshchikov; dateIdentified: 2014; **Event:** eventDate: II-III.2010; **Record Level:** institutionCode: NRM

##### Distribution

Europe.

##### Notes

New for Italy.

#### Scolobatini

Schmiedeknecht, 1911

#### Scolobates auriculatus

(Fabricius, 1804)

##### Materials

- **Type status:** Other material. **Occurrence:** recordedBy: G. Chessa; individualCount: 1; sex: male; **Location:** country: Italy; stateProvince: Sardinia; verbatimLocality: Iglesias, dintorni colonia Beneck; verbatimElevation: 636 m; verbatimLatitude: 39°20'51.45"N; verbatimLongitude: 8°33'55.40"E; **Identification:** identifiedBy: A. Reshchikov; dateIdentified: 2015; **Event:** samplingProtocol: Malaise trap; eventDate: 16-30.V.2006; **Record Level:** institutionCode: NRM

##### Distribution

Holarctic and Indian peninsula.

##### Notes

Already recorded for Italy. It is new for Sardinia.

#### Diacritinae

Townes, 1965

#### Diacritus aciculatus

(Vollenhoven, 1878)

##### Materials

- **Type status:** Other material. **Occurrence:** recordedBy: F. Di Giovanni; individualCount: 1; sex: male; **Location:** country: Italy; stateProvince: Friuli-Venezia Giulia; verbatimLocality: Udine, Precenicco, bosco Bando; verbatimElevation: 10 m; verbatimLatitude: 45°46'42.63"N; verbatimLongitude: 13°03'45.84"E; **Identification:** identifiedBy: F. Di Giovanni; dateIdentified: 2013; **Event:** samplingProtocol: Malaise trap; eventDate: 09-21.VI.2013; **Record Level:** institutionCode: MZUR
- **Type status:** Other material. **Occurrence:** recordedBy: F. Di Giovanni; individualCount: 1; sex: male; **Location:** country: Italy; stateProvince: Friuli-Venezia Giulia; verbatimLocality: Udine, San Giorgio di Nogaro, frazione di Zellina, bosco Boscat; verbatimElevation: 10 m; verbatimLatitude: 45°49'59.51"N; verbatimLongitude: 13°10'03.50"E; **Identification:** identifiedBy: F. Di Giovanni; dateIdentified: 2013; **Event:** samplingProtocol: Malaise trap; eventDate: 09-21.VI.2013; **Record Level:** institutionCode: MZUR
- **Type status:** Other material. **Occurrence:** recordedBy: F. Di Giovanni; individualCount: 2; sex: males; **Location:** country: Italy; stateProvince: Friuli-Venezia Giulia; verbatimLocality: Udine, Porpetto, bosco Sgobitta; verbatimElevation: 15 m; verbatimLatitude: 45°51'13.54"N; verbatimLongitude: 13°11'44.78"E; **Identification:** identifiedBy: F. Di Giovanni; dateIdentified: 2013; **Event:** samplingProtocol: Malaise trap; eventDate: 09-21.VI.2013; **Record Level:** institutionCode: MZUR
- **Type status:** Other material. **Occurrence:** recordedBy: F. Di Giovanni; individualCount: 1; sex: male; **Location:** country: Italy; stateProvince: Veneto; verbatimLocality: Treviso, Gaiarine, Francenigo, bosco Crasere; verbatimElevation: 15 m; verbatimLatitude: 45°54'01.85"N; verbatimLongitude: 12°30'00.86"E; **Identification:** identifiedBy: F. Di Giovanni; dateIdentified: 2013; **Event:** samplingProtocol: Malaise trap; eventDate: 10-22.VI.2013; **Record Level:** institutionCode: MZUR
- **Type status:** Other material. **Occurrence:** recordedBy: F. Di Giovanni; individualCount: 4; sex: 3 males, 1 female; **Location:** country: Italy; stateProvince: Veneto; verbatimLocality: Treviso, Gaiarine, Francenigo, bosco Otello; verbatimElevation: 20 m; verbatimLatitude: 45°51'38.06"N; verbatimLongitude: 12°29'31.71"E; **Identification:** identifiedBy: F. Di Giovanni; dateIdentified: 2013; **Event:** samplingProtocol: Malaise trap; eventDate: 10-22.VI.2013; **Record Level:** institutionCode: MZUR
- **Type status:** Other material. **Occurrence:** recordedBy: F. Di Giovanni; individualCount: 1; sex: male; **Location:** country: Italy; stateProvince: Veneto; verbatimLocality: Venezia, Portogruaro, frazione di Lison, bosco del Merlo; verbatimElevation: 0 m; verbatimLatitude: 45°44'58.18"N; verbatimLongitude: 12°44'37.58"E; **Identification:** identifiedBy: F. Di Giovanni; dateIdentified: 2013; **Event:** samplingProtocol: Malaise trap; eventDate: 10-22.VI.2013; **Record Level:** institutionCode: MZUR
- **Type status:** Other material. **Occurrence:** recordedBy: F. Di Giovanni; individualCount: 2; sex: males; **Location:** country: Italy; stateProvince: Veneto; verbatimLocality: Venezia, Portogruaro, frazione di Lison, bosco del Merlo; verbatimElevation: 10 m; verbatimLatitude: 45°44'47.91"N; verbatimLongitude: 12°44'30.40"E; **Identification:** identifiedBy: F. Di Giovanni; dateIdentified: 2013; **Event:** samplingProtocol: Malaise trap; eventDate: 10-22.VI.2013; **Record Level:** institutionCode: MZUR
- **Type status:** Other material. **Occurrence:** recordedBy: F. Di Giovanni; individualCount: 12; sex: 9 males, 3 females; **Location:** country: Italy; stateProvince: Lombardy; verbatimLocality: Mantova, Marmirolo, Bosco della Fontana; verbatimElevation: 35 m; verbatimLatitude: 45°12'00.16"N; verbatimLongitude: 10°44'38.78"E; **Identification:** identifiedBy: F. Di Giovanni; dateIdentified: 2013; **Event:** samplingProtocol: Malaise trap; eventDate: 11-23.VI.2013; **Record Level:** institutionCode: MZUR
- **Type status:** Other material. **Occurrence:** recordedBy: F. Di Giovanni; individualCount: 1; sex: female; **Location:** country: Italy; stateProvince: Veneto; verbatimLocality: Treviso, Gaiarine, Francenigo, bosco Otello; verbatimElevation: 20 m; verbatimLatitude: 45°51'38.06"N; verbatimLongitude: 12°29'31.71"E; **Identification:** identifiedBy: F. Di Giovanni; dateIdentified: 2013; **Event:** samplingProtocol: Malaise trap; eventDate: 23.VII-05.VIII.2013; **Record Level:** institutionCode: MZUR

##### Distribution

Palaearctic.

##### Notes

**Figure 10.**
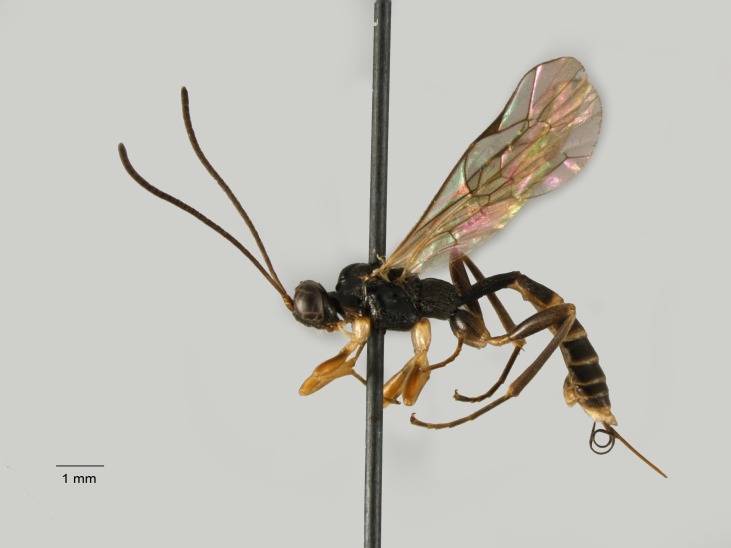
*Diacritus aciculatus* (Vollenhoven, 1878), female.

New for Italy. The subfamily Diacritinae is recorded for the first time for the Italian fauna (Fig. 10​).

#### Diplazontinae

Viereck, 1918

#### Diplazon parvus

Klopfstein, 2014

##### Materials

- **Type status:** Other material. **Occurrence:** individualCount: 1; sex: female; **Location:** country: Italy; stateProvince: Lombardy; verbatimLocality: Mantova, Marmirolo, Bosco della Fontana; verbatimElevation: 35 m; verbatimLatitude: 45°12'00.16"N; verbatimLongitude: 10°44'38.78"E; **Identification:** identifiedBy: F. Di Giovanni; dateIdentified: 2014; **Event:** samplingProtocol: Malaise trap; eventDate: 13-27.V.2008; **Record Level:** institutionCode: MZUR

##### Distribution

Palaearctic.

##### Notes

Recently described by Klopfstein (2014), it can be separated by similar *D. tibiatorius* (Thunberg, 1824) for the smaller size and different sculpture of both mesopleuron and tergites 2 and 3. It is new for Italy.

#### Ichneumoninae

Latreille, 1802

#### Heresiarchini

Ashmead, 1900

#### Heresiarches eudoxius

(Wesmael, 1845)

##### Materials

- **Type status:** Other material. **Occurrence:** recordedBy: F. Di Giovanni; individualCount: 1; sex: male; **Location:** country: Italy; stateProvince: Friuli-Venezia Giulia; verbatimLocality: Udine, Palazzolo dello Stella, Nogali Braide, bosco Brussa; verbatimElevation: 0 m; verbatimLatitude: 45°45'54.05"N; verbatimLongitude: 13°04'52.15"E; **Identification:** identifiedBy: M. Riedel; dateIdentified: 2014; **Event:** samplingProtocol: Malaise trap; eventDate: 08-18.V.2013; **Record Level:** institutionCode: MZUR
- **Type status:** Other material. **Occurrence:** recordedBy: F. Di Giovanni; individualCount: 1; sex: male; **Location:** country: Italy; stateProvince: Friuli-Venezia Giulia; verbatimLocality: Udine, Porpetto, bosco Sgobitta; verbatimElevation: 15 m; verbatimLatitude: 45°51'13.54"N; verbatimLongitude: 13°11'44.78"E; **Identification:** identifiedBy: M. Riedel; dateIdentified: 2014; **Event:** samplingProtocol: Malaise trap; eventDate: 08-18.V.2013; **Record Level:** institutionCode: MZUR
- **Type status:** Other material. **Occurrence:** recordedBy: F. Di Giovanni; individualCount: 1; sex: male; **Location:** country: Italy; stateProvince: Veneto; verbatimLocality: Treviso, Cessalto, Santa Maria di Campagna, bosco San Marco; verbatimElevation: 0 m; verbatimLatitude: 45°42'21.22"N; verbatimLongitude: 12°34'42.45"E; **Identification:** identifiedBy: M. Riedel; dateIdentified: 2014; **Event:** samplingProtocol: Malaise trap; eventDate: 10-22.VI.2013; **Record Level:** institutionCode: MZUR
- **Type status:** Other material. **Occurrence:** recordedBy: F. Di Giovanni; individualCount: 7; sex: males; **Location:** country: Italy; stateProvince: Friuli-Venezia Giulia; verbatimLocality: Udine, Palazzolo dello Stella, Nogali Braide, bosco Brussa; verbatimElevation: 0 m; verbatimLatitude: 45°45'54.05"N; verbatimLongitude: 13°04'52.15"E; **Identification:** identifiedBy: M. Riedel; dateIdentified: 2014; **Event:** samplingProtocol: Malaise trap; eventDate: 21.VII-03.VIII.2013; **Record Level:** institutionCode: MZUR
- **Type status:** Other material. **Occurrence:** recordedBy: F. Di Giovanni; individualCount: 1; sex: male; **Location:** country: Italy; stateProvince: Veneto; verbatimLocality: Treviso, Gaiarine, Francenigo, bosco Crasere; verbatimElevation: 15 m; verbatimLatitude: 45°54'01.85"N; verbatimLongitude: 12°30'00.86"E; **Identification:** identifiedBy: M. Riedel; dateIdentified: 2014; **Event:** samplingProtocol: Malaise trap; eventDate: 23.VII-05.VIII.2013; **Record Level:** institutionCode: MZUR
- **Type status:** Other material. **Occurrence:** recordedBy: F. Di Giovanni; individualCount: 7; sex: males; **Location:** country: Italy; stateProvince: Veneto; verbatimLocality: Treviso, Cessalto, Santa Maria di Campagna, bosco San Marco; verbatimElevation: 0 m; verbatimLatitude: 45°42'21.22"N; verbatimLongitude: 12°34'42.45"E; **Identification:** identifiedBy: M. Riedel; dateIdentified: 2014; **Event:** samplingProtocol: Malaise trap; eventDate: 23.VII-05.VIII.2013; **Record Level:** institutionCode: MZUR

##### Distribution

Europe.

##### Notes

New for Italy.

#### Ichneumonini

Latreille, 1802

#### Barichneumon montgator

Selfa & Anento, 1996

##### Materials

- **Type status:** Other material. **Occurrence:** recordedBy: D. J. Inclán; individualCount: 1; sex: female; **Location:** country: Italy; stateProvince: Tuscany; verbatimLocality: Siena, San Giovanni d'Asso; verbatimElevation: 285 m; verbatimLatitude: 43°08'13.85"N; verbatimLongitude: 11°33'50.53"E; **Identification:** identifiedBy: M. Riedel; dateIdentified: 2014; **Event:** samplingProtocol: yellow pan trap; eventDate: 23-26.X.2012; **Record Level:** institutionCode: MR
- **Type status:** Other material. **Occurrence:** recordedBy: D. J. Inclán; individualCount: 1; sex: female; **Location:** country: Italy; stateProvince: Tuscany; verbatimLocality: Siena, San Quirico d'Orcia; verbatimElevation: 285 m; verbatimLatitude: 43°04'30.84"N; verbatimLongitude: 11°34'44.84"E; **Identification:** identifiedBy: M. Riedel; dateIdentified: 2014; **Event:** samplingProtocol: yellow pan trap; eventDate: 23-26.X.2012; **Record Level:** institutionCode: MR

##### Distribution

Previously known only after the original description from Spain (Selfa and Anento 1996).

##### Notes

New for Italy.

#### Cratichneumon albifrons

(Stephens, 1835)

##### Materials

- **Type status:** Other material. **Occurrence:** recordedBy: Haeseler; individualCount: 1; sex: female; **Location:** country: Italy; stateProvince: Sardinia; verbatimLocality: Mt. Limbara, 8 km SE of Tempio (Vallicciola); verbatimElevation: 900 m; **Identification:** identifiedBy: M. Riedel; dateIdentified: 2015; **Event:** eventDate: 19.V.1996; **Record Level:** institutionCode: ZSM

##### Distribution

Europe.

##### Notes

New for Italy (Sardinia).

#### Eutanyacra pallidicornis

(Gravenhorst, 1829)

##### Materials

- **Type status:** Other material. **Occurrence:** recordedBy: F. Di Giovanni; individualCount: 1; sex: male; **Location:** country: Italy; stateProvince: Veneto; verbatimLocality: Treviso, Gaiarine, Francenigo, bosco Otello; verbatimElevation: 15 m; verbatimLatitude: 45°51'40.91"N; verbatimLongitude: 12°29'37.53"E; **Identification:** identifiedBy: M. Riedel; dateIdentified: 2014; **Event:** samplingProtocol: Malaise trap; eventDate: 23.VII-05.VIII.2013; **Record Level:** institutionCode: MZUR

##### Distribution

Europe.

##### Notes

New for Italy.

#### Stenobarichneumon protervus

(Holmgren, 1864)

##### Materials

- **Type status:** Other material. **Occurrence:** recordedBy: F. Di Giovanni; individualCount: 1; sex: female; **Location:** country: Italy; stateProvince: Veneto; verbatimLocality: Treviso, Gaiarine, Francenigo, bosco Otello; verbatimElevation: 20 m; verbatimLatitude: 45°51'38.06"N; verbatimLongitude: 12°29'31.71"E; **Identification:** identifiedBy: M. Riedel; dateIdentified: 2014; **Event:** samplingProtocol: Malaise trap; eventDate: 09-19.V.2013; **Record Level:** institutionCode: MR
- **Type status:** Other material. **Occurrence:** recordedBy: F. Di Giovanni; individualCount: 1; sex: female; **Location:** country: Italy; stateProvince: Friuli-Venezia Giulia; verbatimLocality: Udine, Muzzana del Turgnano; verbatimElevation: 0 m; verbatimLatitude: 45°47'56.80"N; verbatimLongitude: 13°06'36.09"E; **Identification:** identifiedBy: M. Riedel; dateIdentified: 2014; **Event:** samplingProtocol: Malaise trap; eventDate: 21.VII-03.VIII.2013; **Record Level:** institutionCode: MZUR

##### Distribution

Europe.

##### Notes

New for Italy. The specimen from Veneto (bosco Otello) differs from the typical form in having flagellum with 26 flagellomeres and postpetiolus, tergite 6, scutellum and outer orbits black.

#### Vulgichneumon trifarius

(Berthoumieu, 1892)

##### Materials

- **Type status:** Other material. **Occurrence:** recordedBy: F. Di Giovanni; individualCount: 1; sex: male; **Location:** country: Italy; stateProvince: Friuli-Venezia Giulia; verbatimLocality: Udine, Palazzolo dello Stella, Nogali Braide, bosco Brussa; verbatimElevation: 0 m; verbatimLatitude: 45°45'54.05"N; verbatimLongitude: 13°04'52.15"E; **Identification:** identifiedBy: M. Riedel; dateIdentified: 2014; **Event:** samplingProtocol: Malaise trap; eventDate: 08-18.V.2013; **Record Level:** institutionCode: MZUR

##### Distribution

Europe and Middle East.

##### Notes

New for Italy.

#### Phaeogenini

Förster, 1869

#### Aethecerus ruberpedatus

Diller & Shaw, 2014

##### Materials

- **Type status:** Other material. **Occurrence:** recordedBy: F. Di Giovanni; individualCount: 1; sex: female; **Location:** country: Italy; stateProvince: Veneto; verbatimLocality: Treviso, Cessalto, Santa Maria di Campagna, bosco San Marco; verbatimElevation: 0 m; verbatimLatitude: 45°42'21.22"N; verbatimLongitude: 12°34'42.45"E; **Identification:** identifiedBy: E. Diller; dateIdentified: 2014; **Event:** samplingProtocol: Malaise trap; eventDate: 23.VII-05.VIII.2013; **Record Level:** institutionCode: MZUR

##### Distribution

Previously known only from England.

##### Notes

This species was recently described from England (Diller and Shaw 2014). It is new for Italy and the record suggests the species may have a wider distribution in Europe.

#### Diadromus subtilicornis

(Gravenhorst, 1829)

##### Materials

- **Type status:** Other material. **Occurrence:** recordedBy: D. J. Inclán; individualCount: 1; sex: male; **Location:** country: Itay; stateProvince: Tuscany; verbatimLocality: Siena, Vescona Chiesa; verbatimElevation: 315 m; verbatimLatitude: 43°16'30.47"N; verbatimLongitude: 11°29'29.97"E; **Identification:** identifiedBy: E. Diller; dateIdentified: 2014; **Event:** samplingProtocol: yellow pan trap; eventDate: 22-23.VI.2012; **Record Level:** institutionCode: MZUR
- **Type status:** Other material. **Occurrence:** recordedBy: D. J. Inclán; individualCount: 1; sex: female; **Location:** country: Itay; stateProvince: Tuscany; verbatimLocality: Siena, Arbia; verbatimElevation: 220 m; verbatimLatitude: 43°16'53.18"N; verbatimLongitude: 11°25'35.12"E; **Identification:** identifiedBy: E. Diller; dateIdentified: 2014; **Event:** samplingProtocol: yellow pan trap; eventDate: 13-16.IX.2012; **Record Level:** institutionCode: MZUR

##### Distribution

Holarctic.

##### Notes

New for Italy.

#### Dicaelotus (Dicaelotus) morosator

Aubert, 1969

##### Materials

- **Type status:** Other material. **Occurrence:** recordedBy: F. Di Giovanni; individualCount: 1; sex: male; **Location:** country: Italy; stateProvince: Veneto; verbatimLocality: Venezia, Mestre, bosco di Carpenedo; verbatimElevation: 10 m; verbatimLatitude: 45°30'40.61"N; verbatimLongitude: 12°14'46.92"E; **Identification:** identifiedBy: E. Diller; dateIdentified: 2014; **Event:** samplingProtocol: Malaise trap; eventDate: 22.VII-04.VIII.2013; **Record Level:** institutionCode: MZUR
- **Type status:** Other material. **Occurrence:** recordedBy: F. Di Giovanni; individualCount: 1; sex: male; **Location:** country: Italy; stateProvince: Veneto; verbatimLocality: Treviso, Meolo; verbatimElevation: 0 m; verbatimLatitude: 45°36'24.76"N; verbatimLongitude: 12°27'25.19"E; **Identification:** identifiedBy: E. Diller; dateIdentified: 2014; **Event:** samplingProtocol: Malaise trap; eventDate: 23.VII-05.VIII.2013; **Record Level:** institutionCode: MZUR

##### Distribution

France and Turkey.

##### Notes

**Figure 11.**
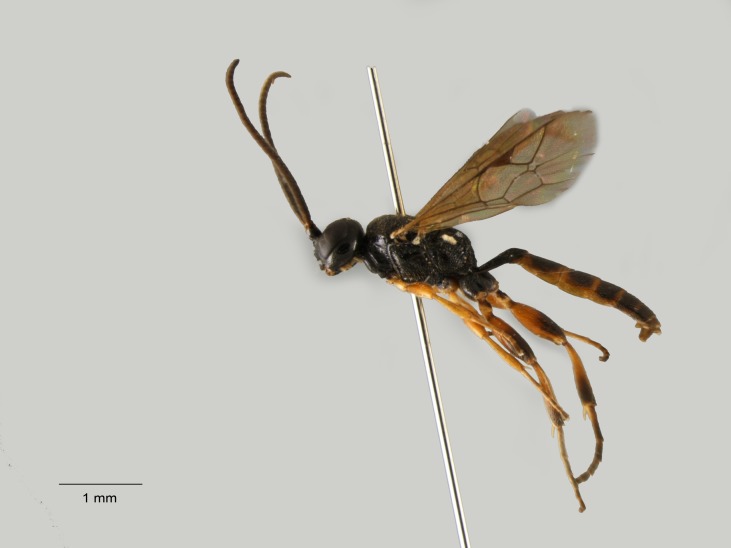
Dicaelotus (Dicaelotus) morosator Aubert, 1969, male.

New for Italy (Fig. 11​).

#### Dicaelotus (Dicaelotus) suspectus

Perkins, 1953

##### Materials

- **Type status:** Other material. **Occurrence:** recordedBy: F. Di Giovanni; individualCount: 1; sex: male; **Location:** country: Italy; stateProvince: Veneto; verbatimLocality: Venezia, Concordia Sagittaria, frazione Sindacale, bosco delle Lame; verbatimElevation: 0 m; verbatimLatitude: 45°41'47.55"N; verbatimLongitude: 12°52'05.79"E; **Identification:** identifiedBy: E. Diller; dateIdentified: 2014; **Event:** samplingProtocol: Malaise trap; eventDate: 21.VII-03.VIII.2013; **Record Level:** institutionCode: MZUR
- **Type status:** Other material. **Occurrence:** recordedBy: F. Di Giovanni; individualCount: 1; sex: female; **Location:** country: Italy; stateProvince: Veneto; verbatimLocality: Treviso, Cessalto, bosco Olmè; verbatimElevation: 10 m; verbatimLatitude: 45°41'55.82"N; verbatimLongitude: 12°37'08.53"E; **Identification:** identifiedBy: E. Diller; dateIdentified: 2014; **Event:** samplingProtocol: Malaise trap; eventDate: 23.VII-05.VIII.2013; **Record Level:** institutionCode: MZUR

##### Distribution

Europe.

##### Notes

New for Italy.

#### Dicaelotus (Gnathichneumon) mandibulator

(Aubert, 1958)

##### Materials

- **Type status:** Other material. **Occurrence:** recordedBy: D. J. Inclán; individualCount: 1; sex: male; **Location:** country: Italy; stateProvince: Tuscany; verbatimLocality: Siena, San Giovanni d'Asso; verbatimElevation: 285 m; verbatimLatitude: 43°08'13.85"N; verbatimLongitude: 11°33'50.53"E; **Identification:** identifiedBy: E. Diller; dateIdentified: 2014; **Event:** samplingProtocol: yellow pan trap; eventDate: 09-10.VI.2012; **Record Level:** institutionCode: MZUR

##### Distribution

Europe and Middle East.

##### Notes

New for Italy.

#### Herpestomus minimus

(Berthoumieu, 1901)

##### Materials

- **Type status:** Other material. **Occurrence:** recordedBy: D. J. Inclán; individualCount: 1; sex: female; **Location:** country: Italy; stateProvince: Tuscany; verbatimLocality: Siena, San Quirico d'Orcia; verbatimElevation: 285 m; verbatimLatitude: 43°04'30.84"N; verbatimLongitude: 11°34'44.84"E; **Identification:** identifiedBy: E. Diller; dateIdentified: 2014; **Event:** samplingProtocol: yellow pan trap; eventDate: 24-25.VII.2012; **Record Level:** institutionCode: MZUR
- **Type status:** Other material. **Occurrence:** recordedBy: F. Di Giovanni; individualCount: 1; sex: male; **Location:** country: Italy; stateProvince: Veneto; verbatimLocality: Treviso, Meolo; verbatimElevation: 0 m; verbatimLatitude: 45°36'24.76"N; verbatimLongitude: 12°27'25.19"E; **Identification:** identifiedBy: E. Diller; dateIdentified: 2014; **Event:** samplingProtocol: Malaise trap; eventDate: 23.VII-05.VIII.2013; **Record Level:** institutionCode: MZUR

##### Distribution

Europe.

##### Notes

New for Italy.

#### Tycherus amaenus

(Wesmael, 1845)

##### Materials

- **Type status:** Other material. **Occurrence:** recordedBy: F. Di Giovanni; individualCount: 1; sex: female; **Location:** country: Italy; stateProvince: Veneto; verbatimLocality: Treviso, Cessalto, Santa Maria di Campagna, bosco San Marco; verbatimElevation: 0 m; verbatimLatitude: 45°42'21.22"N; verbatimLongitude: 12°34'42.45"E; **Identification:** identifiedBy: E. Diller; dateIdentified: 2014; **Event:** samplingProtocol: Malaise trap; eventDate: 10-22.VI.2013; **Record Level:** institutionCode: MZUR

##### Distribution

Europe.

##### Notes

New for Italy.

#### Tycherus juvenilis

(Wesmael, 1848)

##### Materials

- **Type status:** Other material. **Occurrence:** recordedBy: F. Di Giovanni; individualCount: 1; sex: male; **Location:** country: Italy; stateProvince: Veneto; verbatimLocality: Treviso, Gaiarine, Francenigo, bosco Otello; verbatimElevation: 20 m; verbatimLatitude: 45°51'38.06"N; verbatimLongitude: 12°29'31.71"E; **Identification:** identifiedBy: E. Diller; dateIdentified: 2014; **Event:** samplingProtocol: Malaise trap; eventDate: 10-22.VI.2013; **Record Level:** institutionCode: MZUR

##### Distribution

Europe.

##### Notes

New for Italy.

#### Zimmeriini

Heinrich, 1934

#### Cotiheresiarches dirus

(Wesmael, 1853)

##### Materials

- **Type status:** Other material. **Occurrence:** recordedBy: A. Chiocchio; individualCount: 1; sex: female; **Location:** country: Italy; stateProvince: Latium; verbatimLocality: Roma, San Polo dei Cavalieri; **Identification:** identifiedBy: F. Di Giovanni; dateIdentified: 2011; **Event:** eventDate: V.2008; **Record Level:** institutionCode: MZUR

##### Distribution

Europe, North Africa and Middle East.

##### Notes

**Figure 12.**
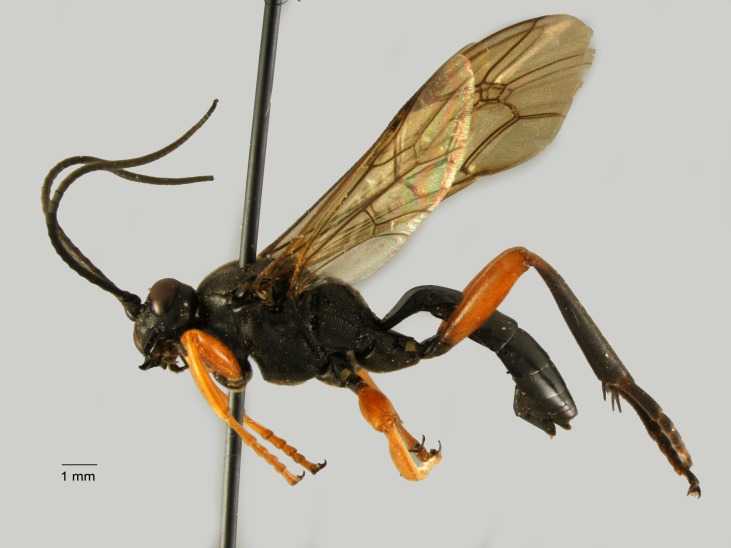
*Cotiheresiarches dirus* (Wesmael, 1853), female.

New for Italy. The tribe Zimmeriini is recorded for the first time for the Italian fauna (Fig. 12​).

#### Lycorininae

Cushman & Rohwer, 1920

#### Lycorina triangulifera

Holmgren, 1859

##### Materials

- **Type status:** Other material. **Occurrence:** recordedBy: F. Di Giovanni; individualCount: 1; sex: male; **Location:** country: Italy; stateProvince: Veneto; verbatimLocality: Treviso, Cessalto, Santa Maria di Campagna, bosco San Marco; verbatimElevation: 0 m; verbatimLatitude: 45°42'21.22"N; verbatimLongitude: 12°34'42.45"E; **Identification:** identifiedBy: F. Di Giovanni; dateIdentified: 2013; **Event:** samplingProtocol: Malaise trap; eventDate: 23.VII-05.VIII.2013; **Record Level:** institutionCode: MZUR

##### Distribution

Palaearctic.

##### Notes

**Figure 13.**
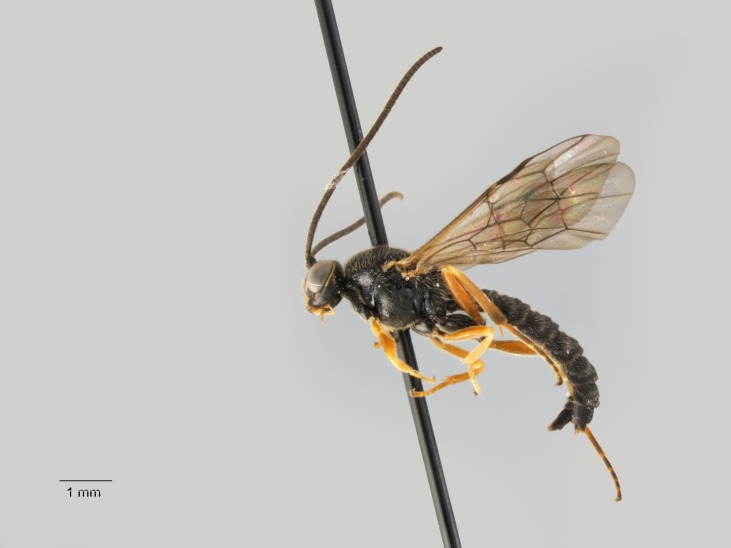
*Lycorina triangulifera* Holmgren, 1859, male.

Already recorded for Sardinia. It is new for the Italian mainland (​​Fig. 13​).

#### Mesochorinae

Förster, 1869

#### Astiphromma aggressor

(Fabricius, 1804)

##### Materials

- **Type status:** Other material. **Occurrence:** individualCount: 1; sex: female; **Location:** country: Italy; stateProvince: Lombardy; verbatimLocality: Mantova, Marmirolo, Bosco della Fontana; verbatimElevation: 35 m; verbatimLatitude: 45°12'00.16"N; verbatimLongitude: 10°44'38.78"E; **Identification:** identifiedBy: M. Riedel; dateIdentified: 2014; **Event:** samplingProtocol: Malaise trap; eventDate: 15-29.IV.2008; **Record Level:** institutionCode: MR
- **Type status:** Other material. **Occurrence:** recordedBy: F. Di Giovanni; individualCount: 1; sex: female; **Location:** country: Italy; stateProvince: Friuli-Venezia Giulia; verbatimLocality: Udine, Muzzana del Turgnano, Selva di Avronchi; verbatimElevation: 10 m; verbatimLatitude: 45°47'28.76"N; verbatimLongitude: 13°07'04.44"E; **Identification:** identifiedBy: M. Riedel; dateIdentified: 2014; **Event:** samplingProtocol: Malaise trap; eventDate: 21.VII-03.VIII.2013; **Record Level:** institutionCode: MR

##### Distribution

Europe.

##### Notes

New for Italy.

#### Astiphromma albitarse

(Brischke, 1880)

##### Materials

- **Type status:** Other material. **Occurrence:** individualCount: 10; sex: 9 males, 1 female; **Location:** country: Italy; stateProvince: Lombardy; verbatimLocality: Mantova, Marmirolo, Bosco della Fontana; verbatimElevation: 35 m; verbatimLatitude: 45°12'00.16"N; verbatimLongitude: 10°44'38.78"E; **Identification:** identifiedBy: M. Riedel; dateIdentified: 2014; **Event:** samplingProtocol: Malaise trap; eventDate: 15-29.IV.2008; **Record Level:** institutionCode: MR
- **Type status:** Other material. **Occurrence:** recordedBy: F. Di Giovanni; individualCount: 1; sex: male; **Location:** country: Italy; stateProvince: Friuli-Venezia Giulia; verbatimLocality: Udine, San Giorgio di Nogaro, bosco Ronchi di Sass; verbatimElevation: 5 m; verbatimLatitude: 45°48'21.10"N; verbatimLongitude: 13°14'27.08"E; **Identification:** identifiedBy: M. Riedel; dateIdentified: 2014; **Event:** samplingProtocol: Malaise trap; eventDate: 08-18.V.2013; **Record Level:** institutionCode: MR

##### Distribution

Europe.

##### Notes

New for Italy.

#### Metopiinae

Förster, 1869

#### Stethoncus sulcator

Aubert, 1963

##### Materials

- **Type status:** Other material. **Occurrence:** individualCount: 1; sex: female; **Location:** country: Italy; stateProvince: Lombardy; verbatimLocality: Mantova, Marmirolo, Bosco della Fontana; verbatimElevation: 35 m; verbatimLatitude: 45°12'00.16"N; verbatimLongitude: 10°44'38.78"E; **Identification:** identifiedBy: F. Di Giovanni; dateIdentified: 2012; **Event:** samplingProtocol: Malaise trap; eventDate: 05-19.VIII.2008; **Record Level:** institutionCode: MZUR

##### Distribution

Palaearctic.

##### Notes

New for Italy.

#### Orthocentrinae

Förster, 1869

#### Batakomacrus flaviceps

(Gravenhorst, 1829)

##### Materials

- **Type status:** Other material. **Occurrence:** recordedBy: G. Chessa; individualCount: 1; sex: female; **Location:** country: Italy; stateProvince: Sardinia; verbatimLocality: Iglesias, dintorni colonia Beneck; verbatimElevation: 636 m; verbatimLatitude: 39°20'51.45"N; verbatimLongitude: 8°33'55.40"E; **Identification:** identifiedBy: F. Di Giovanni; dateIdentified: 2014; **Event:** samplingProtocol: Malaise trap; eventDate: 18.IV-02.V.2006; **Record Level:** institutionCode: MZUR

##### Distribution

Europe.

##### Notes

New for Italy (Sardinia).

#### Pimplinae

Wesmael, 1845

#### Ephialtini

Hellén, 1915

#### Polysphincta longa

Kasparyan, 1976

##### Materials

- **Type status:** Other material. **Occurrence:** recordedBy: F. Di Giovanni; individualCount: 1; sex: female; **Location:** country: Italy; stateProvince: Veneto; verbatimLocality: Treviso, Gorgo al Monticano, frazione di Cavalier, bosco di Cavalier; verbatimElevation: 20 m; verbatimLatitude: 45°45'50.54"N; verbatimLongitude: 12°33'03.95"E; **Identification:** identifiedBy: F. Di Giovanni; dateIdentified: 2014; **Event:** samplingProtocol: Malaise trap; eventDate: 23.VII-05.VIII.2013; **Record Level:** institutionCode: MZUR

##### Distribution

Palaearctic.

##### Notes

The species can be distinguished by closely related *P. boops* Tschek, 1869 by the greater number of antennal flagellomeres and the relatively dense pubescence of the mesoscutum (Fritzén and Shaw 2014). It is new for Italy.

#### Zatypota picticollis

(Thomson, 1888)

##### Materials

- **Type status:** Other material. **Occurrence:** recordedBy: F. Di Giovanni; individualCount: 1; sex: female; **Location:** country: Italy; stateProvince: Friuli-Venezia Giulia; verbatimLocality: Udine, San Giorgio di Nogaro, frazione di Zellina, bosco Boscat; verbatimElevation: 10 m; verbatimLatitude: 45°49'59.51"N; verbatimLongitude: 13°10'03.50"E; **Identification:** identifiedBy: F. Di Giovanni; dateIdentified: 2013; **Event:** samplingProtocol: Malaise trap; eventDate: 21.VII-03.VIII.2013; **Record Level:** institutionCode: MZUR

##### Distribution

Europe.

##### Notes

**Figure 14.**
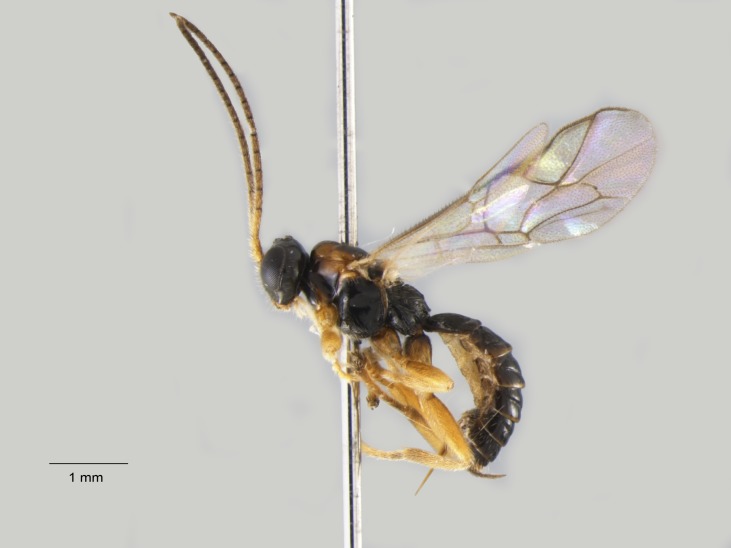
*Zatypota picticollis* (Thomson, 1888), female.

New for Italy (Fig. 14​​).

#### Poemeniinae

Narayanan & Lal, 1953

#### Poemeniini

Narayanan & Lal, 1953

#### Poemenia collaris

(Haupt, 1917)

##### Materials

- **Type status:** Other material. **Occurrence:** recordedBy: F. Di Giovanni; individualCount: 1; sex: female; **Location:** country: Italy; stateProvince: Veneto; verbatimLocality: Venezia, Mestre, bosco di Carpenedo; verbatimElevation: 10 m; verbatimLatitude: 45°30'40.61"N; verbatimLongitude: 12°14'46.92"E; **Identification:** identifiedBy: F. Di Giovanni; dateIdentified: 2013; **Event:** samplingProtocol: Malaise trap; eventDate: 09-21.VI.2013; **Record Level:** institutionCode: MZUR

##### Distribution

Europe.

##### Notes

**Figure 15.**
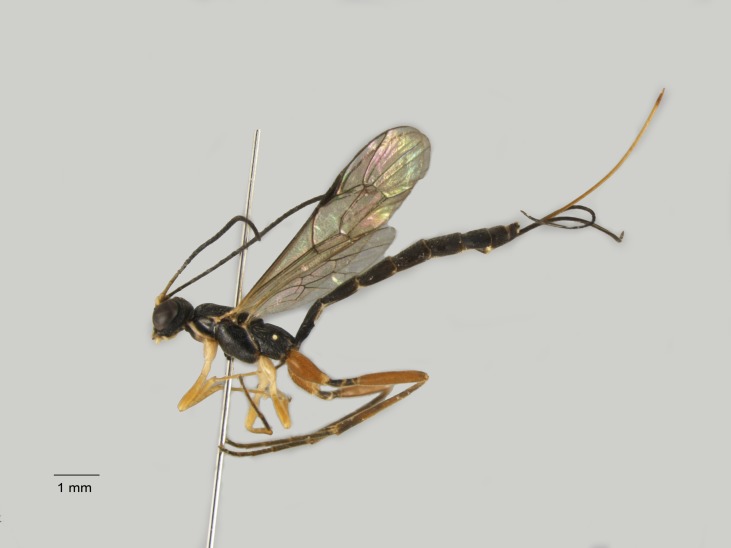
*Poemenia collaris* (Haupt, 1917), female.

New for Italy (Fig. 15​​).

#### Pseudorhyssini

Wahl & Gauld, 1998

#### Pseudorhyssa nigricornis

(Ratzeburg, 1852)

##### Materials

- **Type status:** Other material. **Occurrence:** recordedBy: A. Vigna Taglianti; individualCount: 1; sex: female; **Location:** country: Italy; stateProvince: Piedmont; verbatimLocality: Cuneo, Valle Stura, Sambuco; verbatimElevation: 1200 m; **Identification:** identifiedBy: F. Di Giovanni; dateIdentified: 2014; **Event:** eventDate: 23.VII.1986; **Record Level:** institutionCode: MZUR
- **Type status:** Other material. **Occurrence:** recordedBy: M. Mei; individualCount: 1; sex: female; **Location:** country: Italy; stateProvince: Veneto; verbatimLocality: Belluno, Canale d’Agordo; verbatimElevation: 1000-2000 m; **Identification:** identifiedBy: F. Di Giovanni; dateIdentified: 2014; **Event:** eventDate: 01-26.VII.1995; **Record Level:** institutionCode: MZUR
- **Type status:** Other material. **Occurrence:** recordedBy: L. Colacurcio; individualCount: 1; sex: female; **Location:** country: Italy; stateProvince: Emilia-Romagna; verbatimLocality: Bologna, Rifugio Cavone, Corno alle Scale; verbatimElevation: 1600 m; **Identification:** identifiedBy: F. Di Giovanni; dateIdentified: 2014; **Event:** eventDate: 01.VII.2010; **Record Level:** institutionCode: MZUR

##### Distribution

Holarctic.

##### Notes

New for Italy. The tribe Pseudorhyssini is recorded for the first time for the Italian fauna.

#### Tryphoninae

Shuckard, 1840

#### Tryphonini

Shuckard, 1840

#### Ctenochira meridionator

Aubert, 1969

##### Materials

- **Type status:** Other material. **Occurrence:** recordedBy: G. Chessa; individualCount: 1; sex: female; **Location:** country: Italy; stateProvince: Sardinia; verbatimLocality: Iglesias, dintorni colonia Beneck; verbatimElevation: 636 m; verbatimLatitude: 39°20'51.45"N; verbatimLongitude: 8°33'55.40"E; **Identification:** identifiedBy: A. Reshchikov; dateIdentified: 2015; **Event:** samplingProtocol: Malaise trap; eventDate: 02-16.V.2006; **Record Level:** institutionCode: MR

##### Distribution

Palaearctic.

##### Notes

Already recorded for Italy. It is new for Sardinia.

## Discussion

Most of the data in the present work refer to ichneumonid species that are widespread in Europe or in the Palaearctic region. Besides widespread species, we record some taxa whose geographic ranges are probably confined to the Mediterranean Basin (e.g. *Chirotica rubrotincta*, *Barichneumon montgator*) and others that were previously known only from one or few localities (e.g. *Baranisobas hibericus*, *Agrothereutes monticola*, ​*Phobetes nigriventris*, *Mesoleius tibiator*). For some species (e. g. *Diplazon parvus*, *Dicaelotus morosator*, *Polysphincta longa*), the known distibution should be carefully evaluated, because of possible confusions occurred in the past with morphological similar species (see Aubert 1969, Fritzén and Shaw 2014, Klopfstein 2014).

The present study increases the number of ichneumonids in Italy to more than 2,260 species. Albeit it is a significant improvement over the checklist of 1995 (Scaramozzino 1995​), the comparison with up-to-date checklists of neighbour countries indicates that the list is still largely incomplete: for example, more than 4,000 species have been recorded for Germany, about 3,000 for France and more than 2,500 species for Austria (Yu et al. 2012). In particular, lower attention has been given to the faunistic knowledge of the ichneumonid fauna of southern Italy and its major islands (Scaramozzino 1995). Less than 300 species of ichneumonids are known for Sicily (Turrisi et al. 2007, Riedel and Turrisi 2013, Zwakhals and Turrisi 2014) and only about 150 for Sardinia, a very low number if compared to the about 500 species known for Corsica (Yu et al. 2012).

These data, together with the environmental heterogeneity, resulting from complex orography and latitudinal extension, and the central location of Italy in the Mediterranean region (Minelli et al. 2005​), suggest that the real number of ichneumonids in Italy is much higher than that known today.

## Supplementary Material

XML Treatment for Baranisobas hibericus

XML Treatment for Acaenitinae

XML Treatment for Coleocentrini

XML Treatment for Coleocentrus heteropus

XML Treatment for Adelognathinae

XML Treatment for Adelognathus maculosus

XML Treatment for Banchinae

XML Treatment for Glyptini

XML Treatment for Glypta cylindrator

XML Treatment for Teleutaea striata

XML Treatment for Campopleginae

XML Treatment for Dusona aurita

XML Treatment for Collyriinae

XML Treatment for Collyria trichophthalma

XML Treatment for Cryptinae

XML Treatment for Cryptini

XML Treatment for Agrothereutes leucorhaeus

XML Treatment for Agrothereutes monticola

XML Treatment for Ateleute linearis

XML Treatment for Phygadeuontini

XML Treatment for Chirotica rubrotincta

XML Treatment for Mastrus rufobasalis

XML Treatment for Xenolytus substriatus

XML Treatment for Ctenopelmatinae

XML Treatment for Euryproctini

XML Treatment for Hadrodactylus nigrifemur

XML Treatment for Phobetes nigriventris

XML Treatment for Mesoleiini

XML Treatment for Mesoleius aulicus

XML Treatment for Mesoleius tibialis

XML Treatment for Mesoleius tibiator

XML Treatment for Rhinotorus leucostomus

XML Treatment for Perilissini

XML Treatment for Absyrtus vernalis

XML Treatment for Lathrolestes (Lathrolestes) luteolator

XML Treatment for Lathrolestes (Lathrolestes) tripunctor

XML Treatment for Perilissus pallidus

XML Treatment for Trematopygodes aprilinus

XML Treatment for Pionini

XML Treatment for Rhorus nigrinus

XML Treatment for Sympherta facialis

XML Treatment for Trematopygus lethierryi

XML Treatment for Scolobatini

XML Treatment for Scolobates auriculatus

XML Treatment for Diacritinae

XML Treatment for Diacritus aciculatus

XML Treatment for Diplazontinae

XML Treatment for Diplazon parvus

XML Treatment for Ichneumoninae

XML Treatment for Heresiarchini

XML Treatment for Heresiarches eudoxius

XML Treatment for Ichneumonini

XML Treatment for Barichneumon montgator

XML Treatment for Cratichneumon albifrons

XML Treatment for Eutanyacra pallidicornis

XML Treatment for Stenobarichneumon protervus

XML Treatment for Vulgichneumon trifarius

XML Treatment for Phaeogenini

XML Treatment for Aethecerus ruberpedatus

XML Treatment for Diadromus subtilicornis

XML Treatment for Dicaelotus (Dicaelotus) morosator

XML Treatment for Dicaelotus (Dicaelotus) suspectus

XML Treatment for Dicaelotus (Gnathichneumon) mandibulator

XML Treatment for Herpestomus minimus

XML Treatment for Tycherus amaenus

XML Treatment for Tycherus juvenilis

XML Treatment for Zimmeriini

XML Treatment for Cotiheresiarches dirus

XML Treatment for Lycorininae

XML Treatment for Lycorina triangulifera

XML Treatment for Mesochorinae

XML Treatment for Astiphromma aggressor

XML Treatment for Astiphromma albitarse

XML Treatment for Metopiinae

XML Treatment for Stethoncus sulcator

XML Treatment for Orthocentrinae

XML Treatment for Batakomacrus flaviceps

XML Treatment for Pimplinae

XML Treatment for Ephialtini

XML Treatment for Polysphincta longa

XML Treatment for Zatypota picticollis

XML Treatment for Poemeniinae

XML Treatment for Poemeniini

XML Treatment for Poemenia collaris

XML Treatment for Pseudorhyssini

XML Treatment for Pseudorhyssa nigricornis

XML Treatment for Tryphoninae

XML Treatment for Tryphonini

XML Treatment for Ctenochira meridionator
